# Enterostomy complications in necrotizing enterocolitis (NEC) surgery, a retrospective chart review at Odense University Hospital

**DOI:** 10.1186/s12887-019-1488-5

**Published:** 2019-04-13

**Authors:** Jens Kristian Bælum, Lars Rasmussen, Niels Qvist, Mark Bremholm Ellebæk

**Affiliations:** 1 Surgical Department A, Odense University Hospital, J.B. Winsløws Vej 4, 5000 Odense C, Denmark; 2 OPEN, Odense Patient data Explorative Network, Odense, Denmark

**Keywords:** Necrotizing enterocolitis, Enterostomy, Stenosis, Complications

## Abstract

**Background:**

The aim was to investigate the incidence of postoperative complications to surgery for necrotizing enterocolitis (NEC) with primary focus on enterostomy related complications.

**Methods:**

A retrospective chart review of surgically treated NEC during the period from 2008 to 2014 was performed. Enterostomy with secondary anastomosis was our standard treatment. Postoperative complications were classified according to the Clavien-Dindo Classification (CDC).

**Results:**

Forty-two cases were included in the study. NEC was most frequently located in the small bowel and the length of resected intestine was median 15 cm (2–50). Thirty-nine (93%) patients received an ileostomy and the rest a colostomy. Twenty-two (52%) patients underwent a total of 35 reoperations, and 25 (71%) of these were stoma related with stenosis was the most frequent cause, other causes of reoperation were re-NEC, high-output ileostomy, Ileus and second look.

**Conclusions:**

The rate of reoperation due to complications was high and most often caused by stoma related complications.

## Background

In necrotizing enterocolitis (NEC) surgical intervention is necessary in 20–60% of cases, due to the development of complications such as necrosis of the bowel with or without peritonitis or ileus as the most common [1, 2]. The postoperative period is challenged by complications and prolonged recovery and 20–30% of patients may need further surgery. The postoperative complications include stoma problems, surgical site infections and re-NEC.

Intestinal resection and stoma formation is generally considered as the safest surgical method. But primary anastomosis has proven to be an option, even in extreme low body weight (ELBW) infants [3]. Stoma formation does not always provide a straightforward solution. It involves problems with poor weight gain and electrolyte imbalance due to high intestinal output, stenosis, prolapse, fistula formation and excoriation of the surrounding skin. The metabolic or functional problems may in some cases demand earlier surgery before the planned stoma reversal [4] and may potentially increase the risk of complications.

The objectives of the present study were to:

1) Identify and describe the incidence of postoperative complications in infants operated for NEC with special focus on enterostomy related complications.

2) Identify potential risk factors for reoperation.

3) Evaluate the postoperative complications, occurring up to 1 year after reversal surgery.

## Materials and methods

A retrospective chart review was done, on all patients surgically treated for NEC in the period from 2008 to 01-01 to 2014-12-31 at Odense University hospital (OUH), Denmark, which is a tertiary referral center for pediatric surgery of western Denmark covering approximately 2.6 mill inhabitants.

Exclusion criteria were; no suspicion of NEC during surgery, focal intestinal perforation (FIP), no intestinal resection, no stoma formation, no NEC on histological examination and death within postoperative day 30.

Demographics and postoperative complications were registered. Postoperative complications were classified according to the Clavien-Dindo Classification (CDC). Primary postoperative complications were defined to have occurred between resection and stoma reversal surgery. Secondary postoperative complications were defined as within the first 12 months after stoma reversal surgery.

In this study, NEC was defined as gangrene of intestinal segment with or without perforation and confirmed by histology. In NEC surgery, our standard was a proximal stoma placed in the lower left or right abdominal quadrant with at least 3 cm distance from the abdominal incision. The stoma was fixed by three-point sutures after eversion of the mucosal side, and attempting for a stoma height of approximately 1 cm. The standard for stoma reversal was after 3 months depending upon the child’s clinical condition.

Patients were identified through the ICD-10 diagnosis code DP77.9. Study data were managed using the REDCap (Research Electronic Data Capture) electronic data capture tools hosted by OPEN (Odense Patient data Explorative Network).

Categorical values were presented as median and range. For the comparison between the group of patients with and without complications, a Fisher’s exact test was used.

Data was analyzed using Stata/IC (v14; StataCorp, College Station, Texas), and a *p*-value less than 0.05 was considered as statistical significant.

## Results

**Fig. 1:**
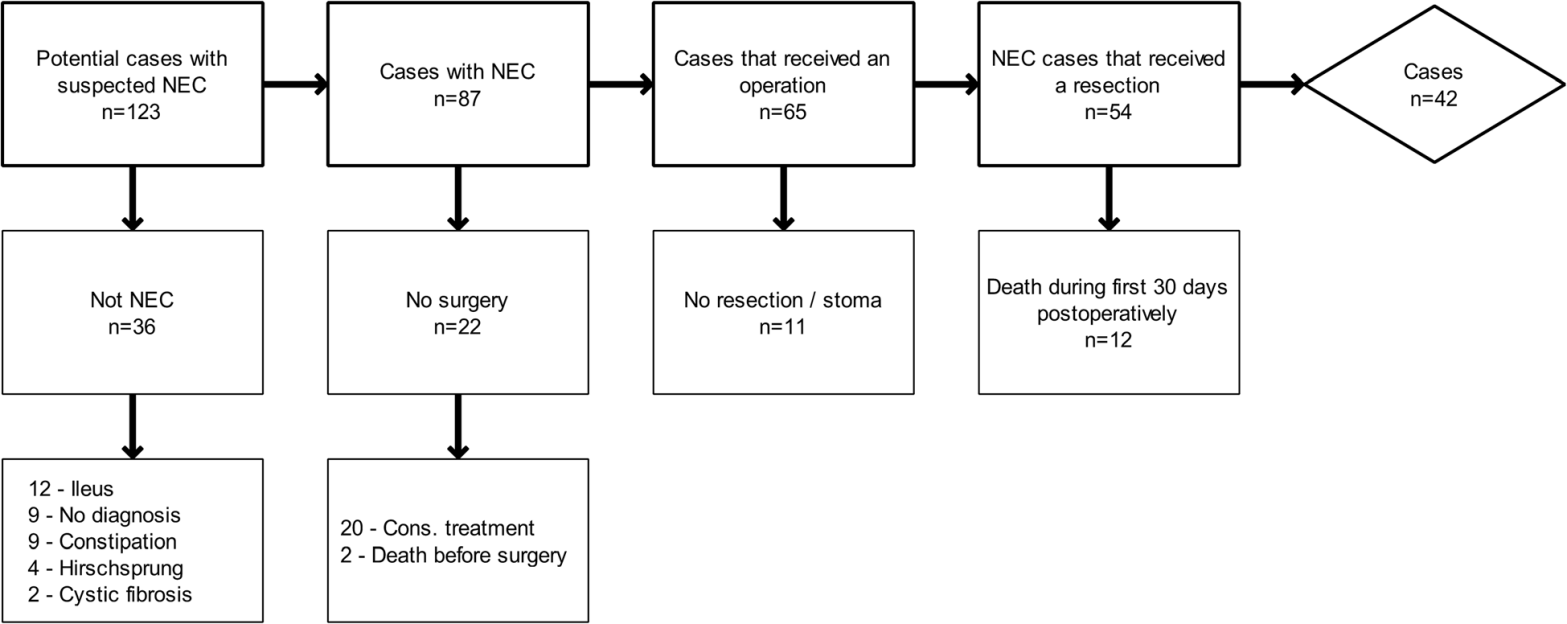
Case inclusion and exclusion flowchart

A total of 123 cases were identified. Thirty-six were excluded, because they did not fulfill NEC criteria, 22 did not undergo surgical treatment. A total of 65 patients underwent laparotomy, 10 did not receive a resection and 12 died within postoperative day 30 not related to stoma complications. Primary anastomosis was chosen in 1 case where a small fibrous segment was resected and histology showed sequela to NEC. For the final analysis 42 cases were included (Fig. 1).

There was an even distribution of gender with 52% male cases (Table 1) and all but one case was born prematurely (before 37 completed weeks of gestation). Low birth weight (< 2500 g) was present in 38 (90%) cases, and extremely low birthweight (< 1000 g) in 18 (43%) cases.

**Table 1:** Characteristics of 42 babies operated for necrotizing enterocolitis (NEC)

Male Sex (%)	22 (52%)
Gestational age in weeks (median + range)	28 (24–37)
Birth weight in grams (median + range)	1028 (553–4810)
Twin (%)	16 (38%)
NEC location (%)	
Small bowel	23 (55%)
Large bowel	13 (31%)
Both	6 (14%)
Resection length in cm (median + range)	15 (2–50)
Perforation with peritonitis	23 (55%)
Type of stoma	
Ileostomy	39 (93%)
Colostomy	3 (7%)
Age at NEC^a^ in days (median + range)	6 (1–55)
Time to reversal^b^ in days (median + range)	124 (34–504)

^a^Time between day of birth and first symptoms of NEC

^b^Time between primary surgery and stoma reversal

NEC was primarily confined to the small bowel (55%). The median length of resected intestine was 15 cm (range 2-50 cm). Perforation with diffuse peritonitis was found in 23 patients (55%). In 39 patients (93%) a small bowel ostomy was performed. The median time from birth to NEC diagnosis was 6 days (range 1–55), and the median time from diagnosis to surgery was 1 day (range 0–13). The median time from primary operation to stoma reversal was 123 days (range 34–504).

A total of 22 (52%) of the babies received a reoperation prior to their planned stoma reversal (CDC ≥ 3b) and in 20 of the babies it was because of a stoma complication (Table 2). The total number of reoperations in the 22 cases was 35 (median 1, range 1–6). Other causes were re-NEC, high stoma output, bowel obstruction and non-planed second look.

**Table 2:** Postoperative and stoma complications in 42 cases of NEC

Cases receiving reoperations	22 (52%)	
Primary cause of reoperation (*n* = 35 in 22 patients)		
Stoma related	25 (71%)	
Re-NEC	6 (17%)	
High-output	2 (6%)	
Ileus	1 (3%)	
Second look	1 (3%)	
	Requiring reoperation	Not requiring reoperation
Stoma related	(*n* = 25 in 20 patients)	(*n* = 10 in 10 patients)
Stenosis	17	0
Fistula	1	3
Ischemia	2	2
Dehiscence	2	0
Retraction	2	0
Prolapse	1	5

The most frequent stoma complication requiring reoperation was stenosis (68%). Other causes were ischemia, dehiscence, retraction, prolapse and fistulation. Ten patients also had stoma-related problems (prolapse, fistulation and ischemia) that did not require surgery (CDC 1–2).

We were not able to show a statistical significant difference in regards to BW, GA, resection length or perforation between the group that received a reoperation and the group that did not.

After stoma reversal 7 cases (16%) had a total of 10 laparotomies, 9 due to bowel obstruction. The last patient had an exploratory laparotomy due to poor weight gain and suspicion of bowel obstruction, but there were no significant pathological findings. All 7 cases had previous reoperations before reversal.

## Discussion

We found a high frequency of post-operative complications requiring surgery. An overall reoperation rate of 52% with the primary cause being stoma related (71%).

The incidence of stoma complications in infants with enterostomies due to NEC has been reported as high as 68%, with a reoperation rate of 25% [5]. The most common complication descripted was enterostomy stenosis, which was present in 40% of cases. The discrepancy between the reoperation rates reported by O’Connor et al., compared to our study might be explained by a more conservative strategy when treating the complications, where more infants were dilated at stoma level or treated with laxatives until the time of the reversal surgery. It is also worth noting, that in the study by O’Connor et al., some strictures were first discovered at the time of reversal, but were still registered as a complication. In our study, stenosis at the time of reversal was not registered as a complication.

In the two cases an earlier reversal operation was performed due to high stoma output, and this was classified as a complication. Refeeding via a mucous fistula was seldom an option as our standard procedure was closure of distal intestinal segment.

The demographic data in our study is comparable to what is reported in the literature on NEC and surgery [3–6] and the 30-day postoperative mortality on 22% within postoperative day 30 is comparable to other studies [5, 7].

The median age of 6 days from birth to onset of NEC symptoms, might be explained by the GA at operation for NEC, which varied from 168 to 260 days. Yee et al. [8] found that higher GA was associated with earlier onset of NEC, meaning that very premature infants will develop NEC late, whereas less premature infants will develop NEC earlier.

Systematic reviews including 10 and 12 studies, respectively, concluded, that there is a general lack of evidence to support primary anastomosis over stoma formation in NEC [9, 10]. The studies included were all retrospective and did not have a clearly defined control group. In all studies, there was a high degree of selection bias, as anastomosis was chosen in the less severe cases of NEC. Haricharan et al. [10] found a significant lower risk of mortality with primary anastomosis in comparison to enterostomy but this might be due to selection bias. The rate of stoma complications was high in our study but this has to be evaluated against the risk of leakage, when choosing primary anastomosis.

When examining the method of stoma formation, it is possible that the eversion method creates ischemia due to the double layering of the intestine, which may lead to stenosis.

Formation of a straight stoma without eversion, might prove superior, as it allows for less stricture of the stoma at skin level. Furthermore, it does not seem that stoma placement in laparotomy incision vs. separate incision affects the risk of complications [11].

Ohashi et al. has proposed a new suture less method of stoma formation in extremely low birth weight infants [12]. In a series of 12 non-NEC cases, there were no serious stoma complications, and only 1 stoma hernia, that did not require surgery.

Time to stoma reversal varied widely from 34 to 504 days. In case of reoperation for any complication before the planned reversal this was registered as a complication. In 7 cases the stoma was closed as there were no contraindications for this (ileus, infection). As for the timing of stoma closure, there is no clear evidence. Veenstra et al. [4]

found no significant difference in morbidity or mortality in early (less than 8 weeks after primary surgery) vs. late closure. Banerjee et al. [13] found that early closure (less than 10 weeks after primary surgery) was associated with longer need of postoperative ventilator support and a higher risk of incisional hernia.

The one-year follow-up period showed that there were significant complications after the reversal surgery. Within the first year, 7 cases (16%) underwent 10 surgeries primary due to obstructive adhesions. All 7 cases also received a reoperation prior to the reversal surgery.

Limitation of the presented study is its retrospective nature, and there is a risk that minor complications were underreported. We were not able collect sufficient data on electrolyte and metabolic imbalances from the patient records. Therefore, these were not included as independent complications in the present study.

We chose to use the Clavien-Dindo classification, as this is the most widely accepted classification for postoperative complications. We are aware of the fact that some of the minor complications might be missed due to poor registration in the patient charts.

Reporting of major complications requiring treatment, on the other hand, were more precise. As the patient population was small, and thus subsequent groups based on risk factors were also small, a risk of type two errors is present. A strength of the present study is that there were no major procedural changes during the inclusion period and all operations were performed by 3 experienced paediatric surgeons.

## Conclusion

In general, there is a high postoperative complication rate in NEC surgery, most complications are related to the stoma and the most frequent is stenosis.

## Abbreviations

BW: Birth weight
CDC: Clavien-Dindo Classification
ELBW: extreme low body weight
FIP: Focal intestinal perforation
GA: Gestational age
NEC: Necrotizing enterocolitis
OPEN: Odense Patient data Explorative Network
OUH: Odense University Hospital
REDCap: Research Electronic Data Capture
